# Bilateral Facial Nerve Palsy and COVID-19 Vaccination: Causation or Coincidence?

**DOI:** 10.7759/cureus.17602

**Published:** 2021-08-31

**Authors:** Matthew C Mason, Adnan Liaqat, Jamie Morrow, Rafaela Basso, Yogesh Gujrati

**Affiliations:** 1 Department of Research, Alabama College of Osteopathic Medicine, Dothan, USA; 2 Internal Medicine, Southeast Health Medical Center, Dothan, USA; 3 Neuro-endovascular Surgery, Southeast Health Medical Center, Dothan, USA

**Keywords:** bell's palsy, bilateral bell's palsy, covid-19, covid-19 vaccine, facial nerve paralysis

## Abstract

Bell’s palsy is a mononeuropathy of the facial nerve that typically causes unilateral facial paralysis. The incidence of unilateral Bell’s palsy is not uncommon, but sequential or simultaneous bilateral Bell’s palsy is exceedingly rare. While unilateral Bell’s palsy is oftentimes idiopathic, bilateral Bell’s palsy is almost exclusively explained by an identifiable trigger. In pre-clinical trials, Bell’s palsy cases were recorded at higher rates in the vaccine cohort than the placebo cohort. Herein, we present a case of isolated sequential bilateral Bell’s palsy that after an extensive workup, proved to be idiopathic. Notably, in the setting of a recent coronavirus disease 2019 (COVID-19) vaccine and absence of identifiable etiology, our case highlights a potential correlation of the COVID-19 vaccine and bilateral Bell’s palsy.

## Introduction

Bell’s palsy (BP) is an acute onset peripheral mononeuropathy of the facial nerve [1]. It is one of the most common acute mononeuropathies with an incidence of 11.5 to 53.3 per 100,000 persons with a strong predilection for females [1,2]. It classically presents as unilateral paralysis with facial weakness, oral insufficiency, altered speech, and an inability to close the eyelids [3]. However, simultaneous or sequential bilateral Bell’s palsy (BBP) is quite rare. Approximately 0.3%-2% of patients with BP will have bilateral disease [4,5]. Thus, the incidence of bilateral disease in the general population is approximately 1 in 5,000,000 [5]. In a meta-analysis of patients with bilateral facial palsy, the age of onset was highly variable and ranged from 8-67 years with an average age of onset of 34 years [6]. 

Unilateral Bell’s palsy is typically a diagnosis of exclusion, and a single etiology has not been identified. Common causes include infection, autoimmune disorders, trauma, vasculitis, cancer, idiopathic, and microvascular ischemia [6-9]. While unilateral BP is oftentimes idiopathic, the bilateral disease is more commonly caused by an identifiable underlying etiology [10].

Cases of BP and BBP have been reported in close proximity to the administration of vaccines, but the literature on the temporal relationship and causation of BP related to coronavirus disease 2019 (COVID-19) vaccine administration is scarce [11]. In the initial data for one manufacturer of the COVID-19 vaccine, four cases of BP were reported in the vaccine cohort and none in the placebo. However, it was reported that the incidence of these cases was consistent with the incidence of the general population. Here, we report a 35-year-old female with isolated, sequential BBP following COVID-19 vaccination, but without clear identifiable etiology. 

## Case presentation

A 35-year-old caucasian female presented with acute, spontaneous onset of right facial weakness and asymmetry with associated occipital headaches and mild right arm weakness. She had difficulty with speech, swallowing, and she was unable to fully close her right eye. She reported no prior history of COVID-19 infection but reported receiving the Moderna mRNA COVID-19 vaccine approximately four weeks prior to the initial presentation. She reported a history of migraine headaches but denied any family or surgical history. She denied home medication. She reported consuming three standard drinks per week but denied tobacco and illicit drug use. Further workup by neuroendovascular surgery included magnetic resonance imaging (MRI) of the brain and computed tomography angiography (CTA) which were unremarkable and negative for ischemic and hemorrhagic stroke. She was initially treated with 500 mg of intravenous (IV) methylprednisolone twice daily for three days and 400 mg of acyclovir four times daily. She was discharged with a tapered prednisone course and advised to complete a 10-day course of 400 mg of acyclovir three times daily for idiopathic BP. 

Four days later, the patient subsequently developed left sided facial weakness, asymmetry, and an inability to close her left eye. She reported a worsening of her dysphagia and dysarthria. During this time, she denied numbness, paresthesias, balance or gait disturbances, vision changes, ptosis, bowel or bladder dysfunction, dizziness, substantial weakness in extremities, and sensory changes. She also denied cough, shortness of breath, joint pain, new onset rashes, tick exposure, chest pain, and palpitations. She was sent for an emergent facial nerve conduction study and electromyography (EMG). Additionally, she was scheduled for lumbar puncture and an extensive serological workup for potential etiologies was performed (see Table 1). 

**Table 1:** Results of cerebral spinal fluid and serological analysis CSF = cerebral spinal fluid; WBC = white blood cell; RBC = red blood cell; ACE = angiotensin converting enzyme; ANA = antinuclear antibody; CRP = C-reactive protein; INR = international normalized ratio; AcH = acetylcholine; N/A = not applicable; CFU = colony forming units; COVID-19 = coronavirus disease 2019

Test	Result	Interpretation	Reference Range	Units
CSF				
Color	Colorless	Normal	Colorless	N/A
Clarity	Clear	Normal	Clear	N/A
Total protein	65	High	15-45	mg/dL
CSF Glucose	73	High	40-70	mg/dL
WBC	0	Normal	0-5	cells/
RBC	6	High	0-2	cells/
ACE	3	Normal	15	U/L
Culture	No growth	Normal	No growth	CFU/mL
Serum				
ANA	Negative	Normal	Negative	N/A
CRP	<0.10	Normal	0-0.99	mg/dL
Sedimentation rate	9	Normal	0-20	mm/hour
INR	1.0	Normal	0.9-1.2	Ratio
AcH receptor	<0.30	Normal	<0.30	nmol/L
Lyme Disease Antibody	<0.90	Normal	<0.90	N/A
Arsenic	<3	Normal	<23	mcg/L
COVID-19	Negative	Normal	Negative	N/A

Physical exam revealed a normal gait and posture with negative Trendelenburg gait and Romberg sign. The patient’s pupils were equal, round, and reactive to light bilaterally. She could not raise her eyebrows or close her eyes bilaterally, and she was unable to frown or smile. Nasolabial fold flattening was noted bilaterally. All other cranial nerve testing was normal. The gross hearing was intact. She had mildly decreased right grip strength, but otherwise, strength testing in the upper and lower extremities was 5/5 on the medical research council for muscle strength. Touch and vibration sensations were intact globally, and her deep tendon reflexes were 2+ in all extremities. 

Nerve conduction and EMG study revealed profound but incomplete axonal loss of the left facial nerve with over 70% axonal loss when compared to the right facial nerve. Lumbar puncture was significant only for a mildly elevated total protein count. Complete blood count, complete metabolic panel, and meningitis panel were unremarkable, as was the contrast MRI of the brain. The differential diagnosis for BBP includes sarcoidosis, Guillain-Barré, Lyme disease, trauma, tumor, and encephalitis; all of which were excluded with serological analyses, cerebral spinal fluid (CSF) analyses, imaging, history of present illness, and physical exam. The patient was continued on 500 mg of IV methylprednisolone twice daily and 400 mg of acyclovir four times daily and achieved significant improvement by day three of admission. The patient was subsequently discharged with neurology outpatient follow-up. 

## Discussion

Unilateral BP is a rather uncommon condition, and in most cases, the etiology is deemed idiopathic in nature. In contrast, BBP is an exceptionally rare condition where an underlying etiology is frequently identified. The most commonly reported causes are Lyme disease, Guillain-Barré syndrome (GBS), and trauma occurring at rates of 36%, 5%, and 4% respectively [10]. Other rare causes of BBP are sarcoidosis and Epstein Barr virus [10]. The underlying pathophysiology is important to tease out as the treatment regimen changes to tailor to an infectious, ischemic, traumatic, or autoimmune etiology. 

Notably, our patient had mildly elevated CSF total protein in the setting of normal WBC’s. Institutional differences in the normal range of total protein are variable, and while our patient had CSF total protein levels at 65 mg/dL with an institutional upper limit of the normal set at 45 mg/dL, some institutions will consider 60 mg/dL as the upper limit of normal. Nevertheless, our patient’s albuminocytologic dissociation was mild. This finding is most commonly associated with GBS. However, our patient exhibited no other signs or symptoms of GBS. There are rarely reported cases of predominant Bell’s palsy or BBP with GBS, but we found no reported cases of Bell’s palsy or BBP in complete isolation [12,13].

Interestingly, our patient received the severe acute respiratory syndrome coronavirus 2 (SARS-CoV-2) vaccine four weeks prior to symptom onset. In Pfizer pre-clinical trials, four patients developed unilateral BP compared with zero cases observed in the placebo cohort [14]. Additionally, the Moderna pre-clinical trials reported three patients with BP compared with zero cases in the placebo cohort [15]. The link of vaccine-induced BP has been identified with the intranasal and parenteral influenza vaccine, influenza H1N1 vaccine, and meningococcal conjugate vaccine [15]. However, subsequent repeat trials of said associations had varying degrees of reproducibility [15]. Post-market surveillance for adverse events of Moderna and Pfizer vaccine-related facial paralysis events had an incidence of 0.6% with a median time to symptom onset of two days [11]. Therefore, given the significant delay between vaccination and onset of symptoms, we believe that our patient’s history of COVID-19 vaccination is less likely to be contributory to the development of her BBP. However, due to a negative exhaustive workup for underlying etiologies and a lack of data on the temporal relationship of COVID-19 vaccines and BBP, a potential correlation may be present. 

While spontaneous symptomatic improvement of BP has been reported, prospective randomized controlled studies have shown improved outcomes with glucocorticoid treatment [16,17]. In severe cases, the addition of antiviral therapy with valacyclovir or acyclovir may improve outcomes [18,19]. Our patient was treated with IV methylprednisolone and acyclovir combination therapy and had significant clinical improvement within three days of treatment initiation.

## Conclusions

Idiopathic bilateral facial nerve palsy is very uncommon. Our patient developed bilateral facial nerve palsy following COVID-19 vaccine and in the setting of a negative exhaustive workup, a correlation may exist between BBP and the COVID-19 vaccine. Further studies are warranted to investigate any potential association between the COVID-19 vaccine and bilateral facial nerve palsy.
